# Optimizing phage therapy with antidefense proteins acquired from the environment

**DOI:** 10.3389/fmicb.2026.1796329

**Published:** 2026-06-03

**Authors:** Ritam Das

**Affiliations:** 1 International Max Planck Research School for Biology and Computation (IMPRS-BAC), Max Planck Institute for Molecular Genetics, Berlin, Germany; 2 Laboratory of Structural Biochemistry, Department of Biology, Chemistry, Pharmacy, Freie Universität Berlin, Berlin, Germany

**Keywords:** antidefense systems, antibiotic-resistance, bacterial immunity, bacteriophages, defense systems, viromics

## Introduction

With an estimated number of 10^31^, bacteriophages are ubiquitous biological entities prevalent in various ecological niches (Hendrix et al., 1999; Mushegian, 2020). These viruses are natural predators of bacteria and pose a persistent threat to their populations (Whitman et al., 1998; Buttimer et al., 2017). Phages typically employ two distinct strategies to maintain and release viral particles, the lysogenic and the lytic cycles. While the lysogenic cycle involves the incorporation of the viral genome within the host, the lytic life cycle of a phage comprises a multi-stage process that, unlike lysogenic, mostly leads to host cell death (Howard-Varona et al., 2017). This inherent efficacy of lysing bacteria in which these viruses multiply has insinuated their use as therapeutics for human infections. So-called “Phage therapy,” using viruses to treat human bacterial infections, is not a recent discovery but dates back to the early 1900s. Back in the year 1915, Frederick Twort observed unusual “glassy and transparent” contaminating spots arising from dead bacteria while propagating vaccinia virus. Later, in 1917, Felix d'Hérelle made similar observations and independently published his findings, coining the term “bacteriophage” (D'Herelle, 2007). In 1921, he successfully administered these viruses for treating dysentery in patients, marking the first recorded use of phages as therapeutics (Sulakvelidze et al., 2001; Dublanchet and Fruciano, 2008). This was followed by several other successful cases of phage therapy, including the treatment of Asiatic cholera in Punjab and Assam (Molina-Quiroz et al., 2023). The therapeutic administration of phages remained popular in several parts of Europe and the erstwhile Soviet Union in the 1920s and 1930s (Summers, 2012). It was the advent of broad-spectrum antibiotics and the ease of their mass production that eclipsed their growing popularity. However, what revolutionized modern medicine has been severely misused (Shah et al., 2023), and we are witnessing a global rise of antibiotic resistance among clinical isolates, with a current forecast of 10 million deaths worldwide by 2050 (Collaborators, 2024).

The rise of antimicrobial resistance, coupled with the high development costs and risks associated with formulating new antibiotics, has prompted a renewed interest in phage therapy (Moon et al., 2025). These viruses can be readily isolated from nature and provide a faster alternative to traditional antimicrobials (Das et al., 2022). Phages with either broad or narrow host ranges are commonly found in diverse ecosystems (Bignaud et al., 2025), a feature that facilitates both generalized and personalized administration of these viruses for therapeutic purposes. In recent years, phage therapy has been performed to treat several critical medical conditions, wherein the prescription of antibiotics was either subeffective or compromised due to bacterial resistance. A personalized phage therapy against multidrug-resistant *Acinetobacter baumannii* strains was performed on a 68-year-old diabetic patient with necrotizing pancreatitis. The patient responded positively to the intravenous and percutaneous administration of the viruses, resulting in the clearance of the bacterial infection, leading to a full recovery (Schooley et al., 2017). Another large-scale personalized phage therapy initiative has been reported by the Belgian consortium, which includes Queen Astrid Military Hospital (QAMH), KU Leuven, and Sciensano. This study involved a retrospective analysis of 100 cases, which demonstrated 77.2% clinical improvement and 61.3% bacterial eradication in patients (Pirnay et al., 2024). The generalized prescription of phages has been equally successful, and still practiced at medical institutes such as the Eliava Phage Therapy Center in Tbilisi, Georgia (Kutateladze and Adamia, 2008), and the Phage Therapy Unit (PTU) of Hirszfeld Institute of Immunology and Experimental Therapy, Wrocław, Poland (Zaczek et al., 2020). Additionally, across various phases, several clinical trials for phage therapy have shown efficacy against difficult-to-treat bacterial infections (Uchechukwu and Shonekan, 2024; Hickson et al., 2025). Targeted Gram-negative pathogens include *Escherichia coli* in double-blind studies using SNIPR001 (Trial ID: NCT05277350) and PreforPro (NCT04511221) phage cocktails, and *Pseudomonas aeruginosa* in cystic fibrosis using the AP-PA02 (NCT04596319) phage cocktail. Among Gram-positive pathogens, the most commonly targeted is *Staphylococcus aureus*, particularly in foot ulcers using TP-102 (NCT04803708) and chronic rhinosinusitis infections using the AB-SA01 phage cocktail (ACTRN12616000002482).

While the reemergence of phage therapy holds promise, there have been instances where it has not succeeded (Zaldastanishvili et al., 2021; Blasco et al., 2023). Key factors reported to contribute to therapeutic failure or lack of response include a limited host range, clearance by the human immune system, and the development of bacterial resistance (Ly-Chatain, 2014). However, an aspect that has received less attention, yet is crucial to the success of phage therapy, is surmounting the activity of bacterial defense systems (Karamlou et al., 2025). An ongoing arms race exists between bacteria and their viruses; this has propelled the former to evolve intricate immune responses. This bacterial defense arsenal is not only complex but also exhibits remarkable diversity, with ongoing discoveries continually unveiling new mechanisms (Bernheim and Sorek, 2020; Millman et al., 2022; Georjon and Bernheim, 2023). Understanding this formidable challenge and the development of robust protocols to address this issue will contribute to the growing success of phage therapy.

## Advancing phage therapy with antidefense proteins from the environment

Bacteria have evolved multiple arsenals to counteract the abundance and diversity of viruses. These prokaryotic immune mechanisms are complex, involving products from a single gene or gene islands (also termed as defense islands), which provide partial or full resistance against phage infections (Yi et al., 2025). Until recent years, bacterial immunity was considered to consist mostly of the restriction-modification (RM) system, CRISPR-Cas, and the toxin-antitoxin systems. However, with the advent of state-of-the-art computational and experimental techniques, the field of bacterial immunity has expanded knowledge about novel antiphage systems (Georjon and Bernheim, 2023). As of now, over a hundred novel systems have been described, revealing a wealth of information about intricate bacterial responses to this evolutionary pressure (Bernheim and Sorek, 2020).

According to an analysis of 21,364 genomes of RefSeq, RM, CRISPR-Cas, and Gabija defense systems are highly abundant in bacteria (Georjon and Bernheim, 2023). The prevalence of these systems highlights their evolutionary advantage in infection management and acts as a potential barrier to successful phage therapy, as they can effectively evade therapeutic phages (Bleriot et al., 2024). Therefore, to enhance the success of phage therapy, it is imperative to focus on novel mechanisms that can circumvent or inhibit these potent bacterial defense systems. The evolutionary arms race has also favored phages, leading to the development of strategies to evade the host's immune response. Phages encode a diverse array of antidefense proteins designed to circumvent or inhibit bacterial defense systems, ensuring successful infection and replication within their hosts (Murtazalieva et al., 2024). Many such antidefense proteins have been identified, particularly against prominent defense systems. For instance, the anti-restriction modification system (RMS) proteins include ArdAB (McMahon et al., 2009) and the Ocr protein from phage T7, which targets Type I RMS (Bandyopadhyay et al., 1985), as well as the injected proteins Ip2 and Ip3 from T4 (Silas et al., 2025) and TifA from phi47 against Type IV RMS (Bullen et al., 2024). Similarly, numerous anti-CRISPR proteins have been discovered and are currently utilized in genetic engineering applications (Kraus and Sontheimer, 2023). Additionally, Antine et al. (2024) reported the structural and functional inhibition capabilities of a phage protein, Gad1, against the Gabija phage defense system (Antine et al., 2024). Beyond the major prokaryotic defense systems, several studies have also uncovered phage antidefense strategies targeting Druantia, Thoeris, Hachiman, and Zorya defense systems, among others (Yirmiya et al., 2024; Costa et al., 2025).

**Figure 1:**
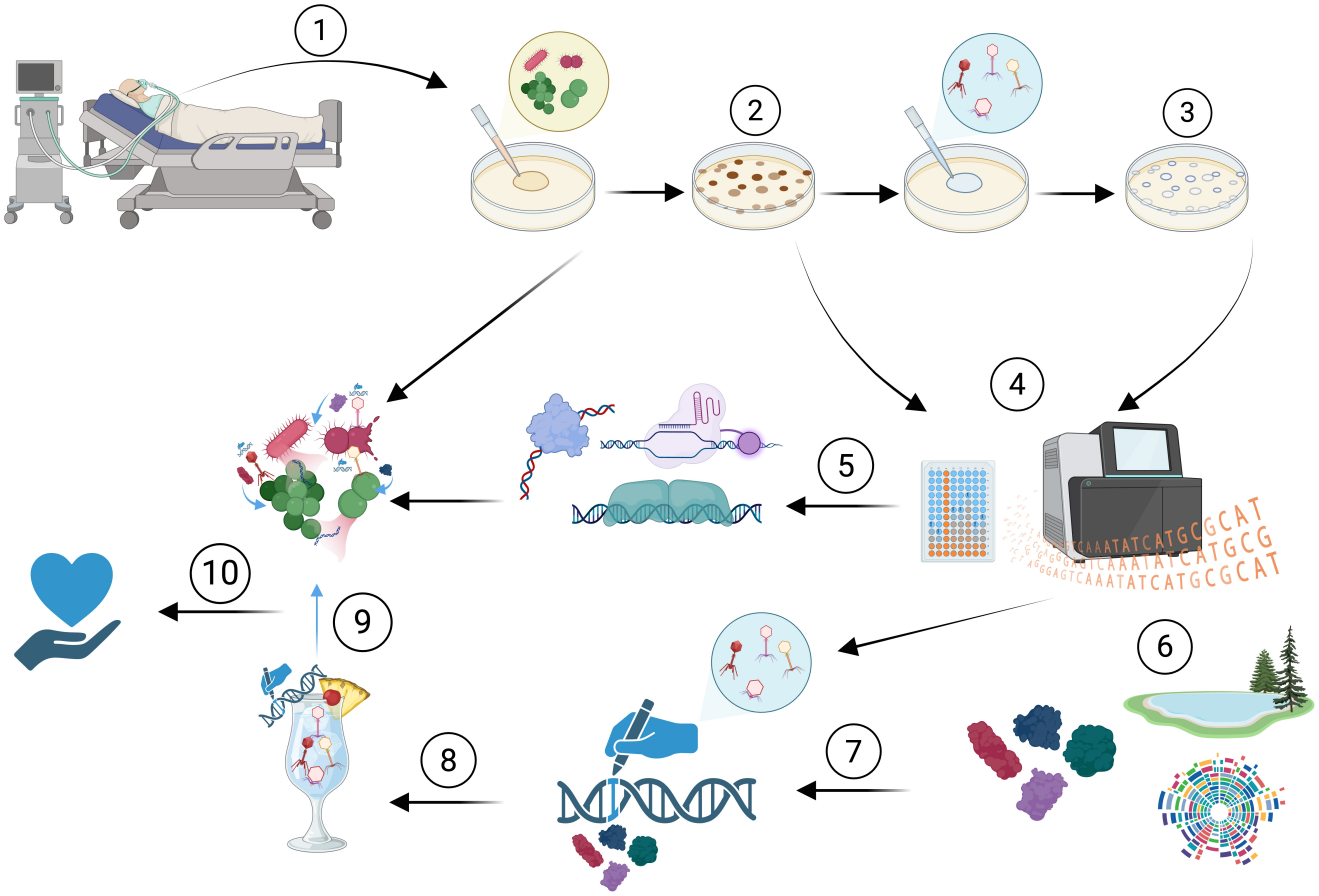
Schematic representation of the advancement of phage therapy with antidefense proteins acquired from the environment. (1) Admission of patient and sample collection. (2) Isolation of causative bacterial pathogens from the patient's samples. (3) Isolation of phages from the environment or testing the activity of available phages from phage banks against the isolated bacterial pathogens. (4) Experimental and genomic characterization of phages and the bacterial pathogens. (5) Identification and characterization of defense system encoded by the bacterial pathogens. (6) Identification and characterization of antidefense systems from metagenomic datasets. (7) Addition of antidefense genes in candidate phage genomes through techniques such as recombineering. (8) Development of engineered phage cocktails for therapeutic applications encoding diverse antidefense systems against the immune systems detected in the bacterial pathogens. (9) Administration of the therapeutic engineered phage cocktail against the disease-causing bacterial pathogens to the patient. (10) Constant monitoring of the patient's vitals, safety markers, immunological responses, and efficacy of the administered phages for therapeutic success. Schematic representation was created in BioRender. Das, R. (2026) https://BioRender.com/6o89qyl.

These antidefense systems are untapped natural resources that can be explored for a variety of purposes. One such prominent field is their employment as therapeutics to target critical phage defense systems in clinically relevant bacterial pathogens that are difficult to treat with antibiotics and phage therapy (Figure 1). Antibiotic-resistant clinical bacterial strains encode multiple defense systems that render phage therapy obsolete (Costa et al., 2024). Resensitizing medically relevant phages by genetically introducing antidefense systems is an effective way to overcome the failure of phage therapy due to bacterial immunity (Yamashita et al., 2025). This approach can be personalized catering to the extent of defense systems a clinical strain from a patient's encoding. Multiple antidefense systems can be introduced into candidate phages through established gene editing techniques, such as Bacteriophage Recombineering of Electroporated DNA (BRED) (Payaslian et al., 2021). Incorporating antidefense genes into phages could play a crucial role in optimizing the phage therapy preparation pipeline, providing an added advantage by enhancing the phages' fitness. This concept has already been tested *in vitro* against Tmn defense system encoded by *E. coli*. A member of the KAP NTPase family, Tmn targets the replication of phages T2, P1, phiVi, and phiX. Identifying and engineering an antidefense protein from ΦSMS22 phage into the Tmn-sensitive ΦKSS9 phage, Yamashita et al. (2025), demonstrated enhanced efficacy in overcoming the Tmn defense, thus highlighting the potential for optimizing phage therapy with antidefense proteins to counteract bacterial defense mechanisms. Defense-guided phage engineering has been demonstrated to be a promising avenue for targeting clinical isolates (Voss et al., 2026). With the introduction of known antidefense systems, a defense-informed phage cocktail prepared by Voss et al. (2026) showcased potency against multi-drug resistant clinical *Staphylococcus aureus* isolates encoding up to 15 known defense systems. Thus, strengthening the concept that phage therapy could be optimized by the exploration and incorporation of antidefense proteins.

## Environmental samples as a source of novel antidefense candidates

Metagenomic profiling of environmental samples is a powerful tool to identify novel biotechnologically relevant proteins (Prayogo et al., 2020). The development of high-throughput facilities for collection, sequencing, and characterization has established the field of viromics (Chang et al., 2024; Roux and Coclet, 2026). This has enabled the study of viruses across diverse environments and has significantly advanced our understanding of the viral “dark matter” (Das et al., 2025; Kosmopoulos and Anantharaman, 2025; Alves et al., 2026). Viromics has also identified several new phage proteins, and this could be the key to the discovery and implementation of novel antidefense proteins. Using a functional metagenomics approach, Rodriguez-Rodriguez et al. (2025) successfully identified over 200 putative defense system components, highlighting the diversity of bacterial immunity. A similar approach can be implemented to study putative antidefense systems from the environment, potentially building a database of antidefense proteins to choose from. Comparable to the isolation and formulation of phage cocktails for therapy, identification and characterization of diverse antidefense proteins from environmental samples to target defense systems in clinical pathogens can be performed. In a clinical setup, phage engineering of antidefense proteins could essentially be an addition to the phage characterization step before therapeutic application, ensuring optimal efficacy of the selected phages (Figure 1).

## Future perspectives

The future of phage therapy, optimized with antidefense proteins from the environment, appears promising. Considering the development of high-throughput gene editing and phage characterization technologies, engineering bacteriophages for critical care patients could be a game-changer in tackling the growing AMR crisis. The reemergence of phage therapy can be a key research area for medical professionals, and tailoring these viruses to the patient's needs can make these therapeutic agents more effective. However, much of the phage genome remains as “encrypted dark matter,” which limits our understanding and repertoire of antidefense proteins that can be potentially used for targeting defense systems in clinical isolates. In this regard, systematic computational and experimental characterization offers a promising approach to advancing phage therapy (Das and Bajpai, 2023; Das et al., 2024). Genome-wide phage random transposon mutagenesis and sequencing have recently been demonstrated to aid the discovery of novel antidefense proteins (Humolli et al., 2025; Kyte et al., 2025). Additionally, synthetically designed proteins have been exhibited to inhibit bacterial defense systems and engineering phages with these *de novo* proteins has provided an improvement in infectivity in bacterial strains carrying multiple defense systems (Garb et al., 2025). In the future, the application of these techniques can expand our arsenal of antidefense measures, particularly against bacterial immune mechanisms that have yet to be discovered.

The AMR conundrum poses a threat to public and animal health, and without proactive strategies, common infections could be life-threatening. Although the concept of targeting bacterial immunity is in its early stages, it offers an encouraging outlook for strengthening phage therapy.
